# High resolution 3D imaging of living cells with sub-optical wavelength phonons

**DOI:** 10.1038/srep39326

**Published:** 2016-12-20

**Authors:** Fernando Pérez-Cota, Richard J. Smith, Emilia Moradi, Leonel Marques, Kevin F. Webb, Matt Clark

**Affiliations:** 1 Optics and photonics group, Faculty of Engineering, University of Nottingham,University Park, Nottingham, UK

## Abstract

Label-free imaging of living cells below the optical diffraction limit poses great challenges for optical microscopy. Biologically relevant structural information remains below the Rayleigh limit and beyond the reach of conventional microscopes. Super-resolution techniques are typically based on the non-linear and stochastic response of fluorescent labels which can be toxic and interfere with cell function. In this paper we present, for the first time, imaging of live cells using sub-optical wavelength phonons. The axial imaging resolution of our system is determined by the acoustic wavelength (*λ*_*a*_ = *λ*_*probe*_/2*n*) and not on the NA of the optics allowing sub-optical wavelength acoustic sectioning of samples using the time of flight. The transverse resolution is currently limited to the optical spot size. The contrast mechanism is significantly determined by the mechanical properties of the cells and requires no additional contrast agent, stain or label to image the cell structure. The ability to breach the optical diffraction limit to image living cells acoustically promises to bring a new suite of imaging technologies to bear in answering exigent questions in cell biology and biomedicine.

Optical microscopy is possibly the most powerful technique used in the life sciences to study cell biology and remains an extremely active global research area, continuing to drive advances in biomedicine. Imaging of live cells is crucial in order to study structure, function and dynamic behaviour – often a difficult problem because of the scale, fragility, and weak optical contrast of living cells. Techniques such as differential interference, phase contrast^1^, epifluorescence^2^ (2D), confocal laser scanning microscopy (CLSM^3^) and multiphoton imaging^4^ are widely used because of their compatibility with live cells. However, living cells are susceptible to photodamage from high photon energies at short wavelengths. This limits the range of wavelengths that can be used to the visible part of the spectrum and ultimately limits the resolution. Super-resolution techniques such as STED^5^ and STORM^6^ can surpass the Rayleigh resolution limit but require high photon doses and fluorescent labels which can be injurious to living preparations^7^. The study of dynamic internal processes involving sub-optical structures (<500 nm) is increasingly important, and there is a limited choice of label-free super-resolution methods capable of addressing living preparations.

Images of individual biological cells using mechanical contrast is of great interest as it can provide complementary information to that obtained with conventional optical microscopy. Current methods for mechanical imaging at optical resolutions or above are not compatible with biological samples, especially living samples. These often require harsh conditions or direct mechanical interactions with the sample which may or may not be destructive or quantitative. Mechanical imaging is currently performed using techniques such as scanning acoustic microscopy (SAM), atomic force microscopy (AFM), photoacoustic microscopy (PAM) and Brillouin microscopy.

SAM^8^,^9^ techniques are usually based on piezoelectric transducers where the attenuation of sound in culture medium limits the propagation distance and hence the resolution to below that of an optical microscope^10^. AFM^11^,^12^ can resolve sub-optical features using a sharp nanoprobe to test the height and rigidity of a surface. While a powerful and diversely-applied method in other fields, AFM is difficult in living cells and is intrinsically sensitive only near the surface of preparations without causing damage. PAM^13^ relies on the absorption of the sample to produce an acoustic wave for imaging. Here the contrast mechanism is mainly optical absorption and the resolution remains optical. For label free imaging of single cells, optical absorption is not uniform and different parts of the cell require different pumping wavelengths. This means not only multiple laser sources but toxic wavelengths in the UV part of the spectrum^14^. To avoid those, external contrast agents can be also used at single wavelength operation^15^. High resolution label free images have been obtained using PAM, however this is not yet viable for living cells^16^. Brillouin microscopy^17^,^18^ measures the frequency shift (Brillouin frequency) of photons that are scattered by phonons to obtain a measure of the sound velocity. This is the same fundamental mechanism to obtain a measurement presented here, however spontaneous Brillouin scattering is an inefficient process (one in every ~10^10^–10^12^ photons contributes to the signal^19^) since it relies on thermally generated phonons within the sample and thus requires high optical fluxes to obtain sufficient SNR that are incompatible with living cells, furthermore the method is intrinsically optically-limited in resolution because the thermal phonons are incoherent.

## Phonons for cell imaging

Phonons can be generated by the absorption of short light pulses by metallic films^20^ and detected by several methods including; the opto-elastic effect, beam deflection, interferometry or Brillouin oscillations^21^. Phonons have been widely applied for the study of hard solid materials, but only recently applied to biological samples^22^,^23^,^24^. The phonon generation/detection methods are based on optical pump-probe techniques^25^. Here the phonons are generated (usually thermoelastically) by a short laser pulse and then probed by a second time delayed laser pulse. Sweeping the time delay facilitates temporal reconstruction of the waveform. This method allows the observation of very high frequency phonons, up to the THz region^26^. Historically the acquisition speed of this method is slow due to the time required to mechanically sweep the time delay. More recently the development of ASOPS laser systems^27^ have allowed a significant increase acquisition speed (see methods).

**Figure 1:**
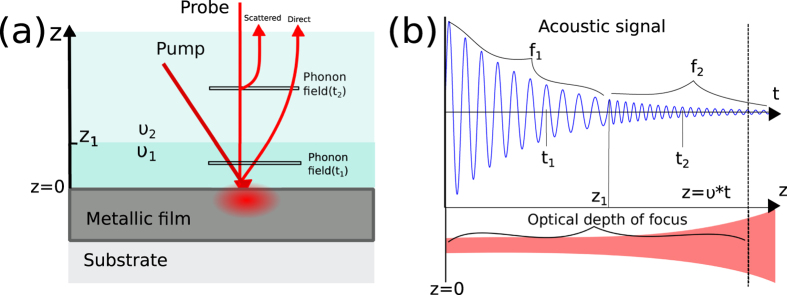
Phononic measurement of transparent materials. In (**a**), a light pulse (pump) is used to thermoelastically generate a coherent phonon field in the metallic film. The phonon field (shown at two positions in time, t_1_ and t_2_, by thin horizontal bars) is probed by a second light beam (probe). The interference of both direct and scattered probe beams induces an oscillation in the detected probe light intensity. The frequency of this oscillation *f*_*B*_ is a function of the speed of sound. As the phonon field travels from one material with speed of sound *ν*_1_ to another material with speed of sound *ν*_2_, the detected frequency changes accordingly as shown in (**b**). As the phonon wavelength is shorter than the optical, it is possible to section the optical volume by post-processing without the need of further acquisition, mechanical positioning or change of focus.

When the sample is translucent, as in the case of biological cells, signals typically known as Brillouin oscillations are observed. These signals arise because there is interference between sound-scattered and directly transmitted or reflected beams (see Fig. 1(a)). The relative phase of the interfering beams changes as the phonon field travels away from the generation film producing a temporal intensity oscillation (see Fig. 1(b)). The frequency of this acoustic signal is the Brillouin frequency, *f*_*B*_ = 2*nν*/*λ*_*probe*_, (normal incidence) where *n* is the refractive index, *ν* the speed of sound and *λ*_*probe*_ the optical probing wavelength. By generating coherent phonons with the same direction and frequency, the random nature of spontaneous generation is avoided increasing the scattering efficiency by several orders of magnitude. For a material with a known refractive index, simple quantitative measurements of the speed of sound are possible. This feature has great potential and it has produced reports in both phononic^28^ and Brillouin microscopy^29^ fields.

The temporal location of the phonons is related to their spatial location through the speed of sound (*z* = *νt*) as shown in Fig. 1(b). Each cycle in the temporal signal corresponds to one acoustic wavelength and hence the Brillouin frequency along the axial direction can be determined with acoustical resolution rather than the optical resolution used to probe the field. Since the acoustical wavelength *λ*_*a*_ = *λ*_*probe*_/2*n* is shorter than the optical, there is the opportunity to axially section the measured optical volume. This could lead to the resolution of objects smaller than the optical depth of focus with the acquisition of a single measurement and without the need of high NA lenses. While the axial resolution can exceed the optical, the generated phonon field is confined in its lateral extent to that of the generation pump wavelength spot setting the lateral resolution to that given by the pump beam wavelength.

Given the potential of high resolution quantitative measurements of a mechanical property such as the speed of sound, Brillouin oscillations has been explored as a cell characterisation tool^22^. Single point phononic measurements of vegetal^30^ and dehydrated^31^ cells have been published using various sample interrogation regimes. Imaging of fixed^24^,^32^ and dehydrated^23^ specimens have also been reported, however the underpinning technologies are incompatible with living specimens. Brillouin oscillation uses short wavelength phonons and long wavelength photons to probe the specimen. The speed of sound is typically 10^5^ times lower than the speed of light. Therefore, for the same wavelength, the frequency and energy carried by a phonon is also 10^5^ times lower compared that of a photons thus eliminating the energetic damage done by short wavelength (UV) photons. Despite this advantage, the damage induced by photons, heat and slow acquisition speeds (due to low SNR) have, until now, rendered phononic live cell imaging impossible.

This paper presents the development and application of an alternative, phonon imaging modality, which overcomes some of the limitations of previous phononic approaches to show, for the first time, the compatibility of phononic imaging with living cells. Additionally, this method can in principle, by working in the GHz regime, generate coherent phonon fields at sub-optical wavelengths. Such wavelengths can be used to extract information in the axial direction below the optical Rayleigh limit. This potential is explored and the axial resolution achievable with this method is analysed.

## Results

**Figure 2:**
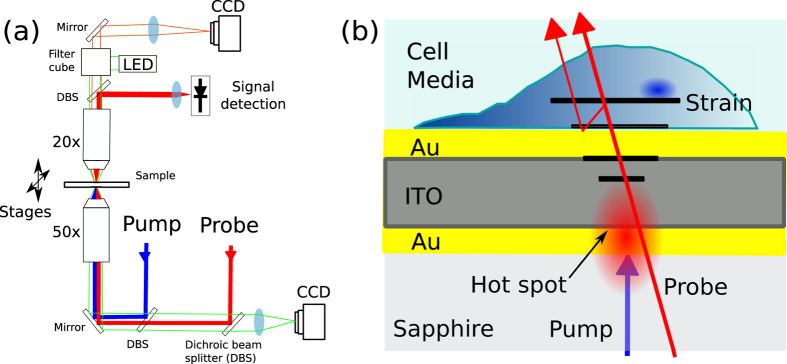
Experimental schematic. (**a**) Experimental setup. Two pulsed lasers in ASOPS pump-probe configuration are combined by an objective lens (0.55 NA) and focused into the transducer substrate interface where the probe beam is captured by a second objective (0.42 NA) and detected in a photodiode. The system is built around a microscope to enable complementary optical imaging. A fluorescence detection arrangement allows the state of a cell to be assessed. (**b**) Transducer and sample arrangement. The pump beam is absorbed while the probe beam is transmitted by the transducer (with dimensions Au = 20 nm, ITO = 140 nm and Au = 20 nm) to allow detection while protecting the cell from optical exposure. The substrate is sapphire to prevent temperature rise.

Our solution to the current barriers to live-cell phononic imaging comprises a series of experimental design choices to minimise exposure of the sample to injurious photon or thermal doses. It uses a novel illumination geometry, novel transducer design, and thermally-conductive substrate to shield and manage the light and thermal exposures. A simplified schematic of our system is presented in Fig. 2. The optical pump (390 nm, ~0.5 mW) and optical probe (~780 nm, 1 mW) beams approach the sample through the substrate, which is selected for minimal optical exposure and optimal thermal dissipation. An opto-acoustic transducer fabricated on top of the substrate consists of an optical cavity composed of three layers. The cavity is precisely designed to strongly absorb the pump light (for phonon generation) while allowing high transmission of the probe beam (for phonon detection). The design parameters of this novel transducer are tuned to produce mechanical as well as optical resonances in the desired performance bands. This configuration thus offers several key advantages over other phonon detection methods: significantly reduced exposure of the sample to both pump and probe beams, increase in signal amplitude through mechanical resonance within the transducer and enhanced thermal management by the use of a high-thermal-conductivity substrate (sapphire). These advantages manifest as greatly improved signal-to-noise ratio (SNR) and minimised thermal disruption to the sample. Increased SNR may be traded for acquisition speed allowing, in our pump-probe ASOPS configuration, acquisition of approximately 5000 traces per second which are averaged to obtain SNR of ~40–70, depending on input power, substrate material and transducer characteristics^24^,^33^. This SNR is sufficient to to image the sample at each point in the x,y dimensions and from the acquired traces, axial information is extracted.

### Sectioning and resolution

Resolving the Brillouin frequency in time gives the possibility of sectioning the optical volume with acoustic resolution. Each cycle of the detected signal corresponds spatially to one acoustic wavelength which is typically shorter than the optical wavelength. By calculating the Brillouin frequency for a small number of cycles, the optical volume can be sectioned. From a theoretical point of view, at least one cycle is necessary to measure the frequency of a sinusoidal function. However in practice this is difficult to achieve. In this section, the axial resolution achievable by processing the Brillouin signals with the short time Fourier transforms (STFT)^34^,^35^ method is analysed based on modelling and single edge experimental targets.

**Figure 3:**
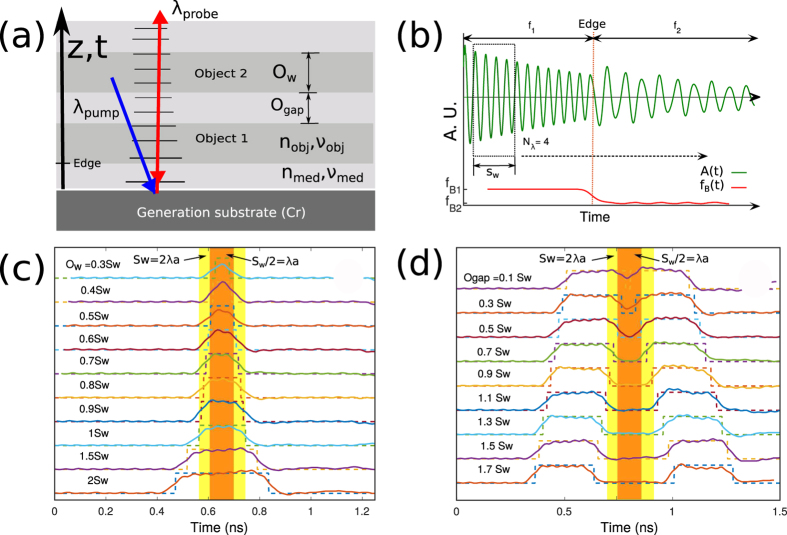
Simulated measurements and axial response upon stimulation. (**a**) Schematic of the simulated geometry. (**b**) Simulation of an edge response to represent sectioning. (**c**) Simulated response from a single object with various sizes for *N*_*λ*_ = 2. (**d**) Simulated response by two near objects at various edge to edge distances using *N*_*λ*_ = 2. Resolution in both cases is half of the measuring window *S*_*w*_/2.

Based on a theoretical thermo-elastic model^36^ (see supplementary information), the generation, propagation of sound and its interaction with light (at *λ*_*probe*_) was simulated for one dimensional space (z). The simulation considered one or two objects immersed in a medium (see Fig. 3(a)) where the width (O_*w*_) and separation of the objects (O_*gap*_) was varied to observe the optical response. The time of flight of the signals can be converted to axial position z. The resultant temporal variation of the simulated light intensity was then post-processed for sectioning. Based on the STFT method, *f*_*B*_ was calculated against time (see Fig. 3(b)). In this method, each window lasted for a few cycles of the Brillouin signal and each cycle of the signal spatially corresponds to an acoustic wavelength *λ*_*a*_ = *λ*_*probe*_/2*n*. The width of each section in spatial units is given by *S*_*w*_ = *λ*_*a*_*N*_*λ*_ where *N*_*λ*_ is the processing window width and is selected to match an integer number of cycles greater or equal than one (see Fig. 3(b)).

Figure 3 shows a simulation of optical sectioning using phonons. Figure 3(a) shows the geometry of the model. The pump wavelength (*λ*_*pump*_ = 390 nm) is absorbed in a metallic film. The generated sound then propagates in the z direction and induces Brillouin oscillations for the probe wavelength (*λ*_*probe*_ = 780 nm). Figure 3(c) shows the response to a single object immersed in medium. The simulated object is made up of layers of pseudo-materials with slightly different mechanical but identical optical properties. In Figs 3(c) and 3(d) the dotted lines represent the ideal response expected from the object and the solid lines show the response measured from the simulations. The width of the section S_*w*_ and a half of the section S_*w*_/2 (for *N*_*λ*_ = 2) are shown as yellow and orange areas respectively. As the object becomes smaller, the response changes until the measured Brillouin frequency of the object does not reach the expected frequency value calculated from the known object properties. This is because the first edge is not fully resolved before the second edge of the object arrives. However, the object is still visible. Here we shall arbitrarily define a resolved object by this method is that one whose measured frequency is within 5% of the expected value. From this definition it was observed that the width of a resolvable object is typically around half the width of the window S_*w*_ (see Fig. 3(c)). For the case of two objects separated by a gap (see Fig. 3(d)), the minimum resolved gap is also half of the section width (since the edge response remains the same). As the object becomes smaller than this, the measured frequency shift is reduced - equivalent to a loss of contrast in optical imaging. If the quantitative measurement of the frequency is not of interest and all that matters is contrast, then relaxing the resolution requirement allows the observation of objects of a quarter of the measuring window (analogous to Sparrow criteria in optics^37^).

The extracted *f*_*B*_ in Fig. 3c,d shows artifacts in the form of ripples, these arise for two main reasons. When the sectioning window, which is fixed in size, transitions from one object to the other, its size is no longer a complete number of cycles producing false phase transitions. To reduce this effect the time trace is windowed (typically by a Hann window) to smooth the edges. Applying this process to a single cycle introduces significant distortions (which limits *N*_*λ*_ > 1). These effects are reduced as *N*_*λ*_ increases.

**Figure 4:**
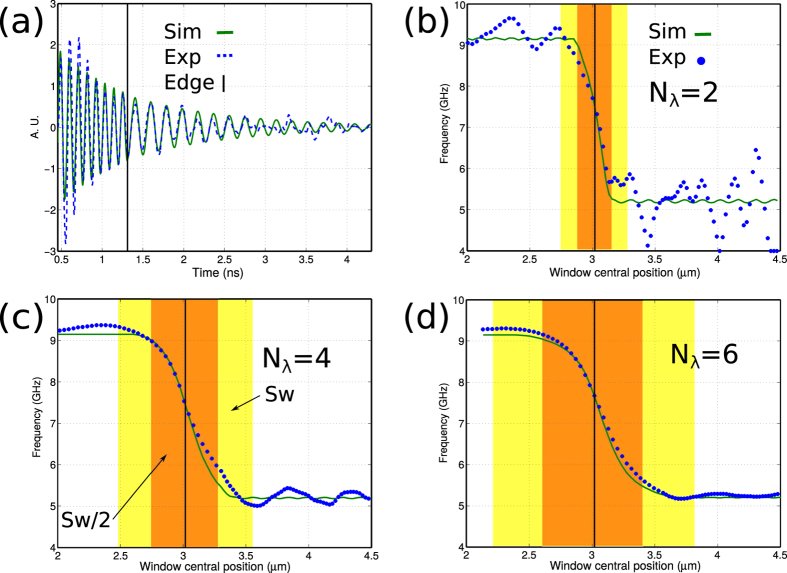
Experimental measurement of edge response between polystyrene and water. (**a**) Experimental and simulated (fitted) traces with a sharp edge made out of polystyrene-water transition. (**b**) Edge response with *N*_*λ*_ = 2. (**c**) Edge response with *N*_*λ*_ = 4. (**d**) Edge response with *N*_*λ*_ = 6. In all *N*_*λ*_ cases the resolution is half of the section width.

A resolution target to experimentally demonstrate the z resolution of this method is difficult to fabricate and characterise accurately, however the response to an edge can confirm the observations from the model. Figure 4 shows the experimental response to an edge made out of polystyrene and water. Experimental and simulated (fitted) variations in intensity are shown in Fig. 4(a). In Fig. 4(b–d), both simulated and experimental signals are processed by STFT using *N*_*λ*_ = 2, 4 and 6 respectively. The experimental response to the edge follows well the simulations confirming that the edge is resolved within half the width of the section. In Fig. 4(b), the ripples in the a simulated trace (artifacts) are smaller to the variations caused by noise. For *N*_*λ*_ = 4 and 6, the ripples are no longer visible (see Fig. 4(c,d)).

The axial resolution obtained using the method presented here has been observed to be approximately half the width of each section *S*_*w*_/2. A fixed number of acoustic cycles *N*_*λ*_ is used in the STFT process where a minimum of *N*_*λ*_ = 2 has been observed as viable. The size of each section is given by *S*_*w*_ = *N*_*λ*_*λ*_*a*_ which gives a simple expression for resolution:


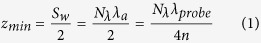


where *λ*_*probe*_ is the optical probing wavelength and *n* the refractive index. The frequency resolution, which governs the smallest detectable variation in *f*_*B*_ is directly proportional to N_*λ*_ and inversely proportional to the axial resolution. Which means that in a practical situation, a trade must be made between frequency and axial resolutions; high frequency resolution will allow the detection of wide objects with weak frequency contrast while high axial resolution of thinner objects will require stronger frequency contrast to be viable. This trade is observable in Fig. 4 where the shorter window shows greater influence from noise than the longer ones.

In practise imaging cells, the thinnest possible section (*N*_*λ*_ = 2) gives the resolution equals to one *λ*_*a*_ ~ 280 nm at 780 nm. However, due to the presence of noise, the number of acoustic cycles *N*_*λ*_ used for the sectioning windows in cell imaging is typically four to six reducing the axial resolution. For *N*_*λ*_ = 4 or 6, the resolution is 560 and 840 nm which can only be matched by confocal microscopy using very high NA (~1.7) objective lens, whereas a 0.42 NA lens is used in this work.

### Phononic cell imaging

**Figure 5:**
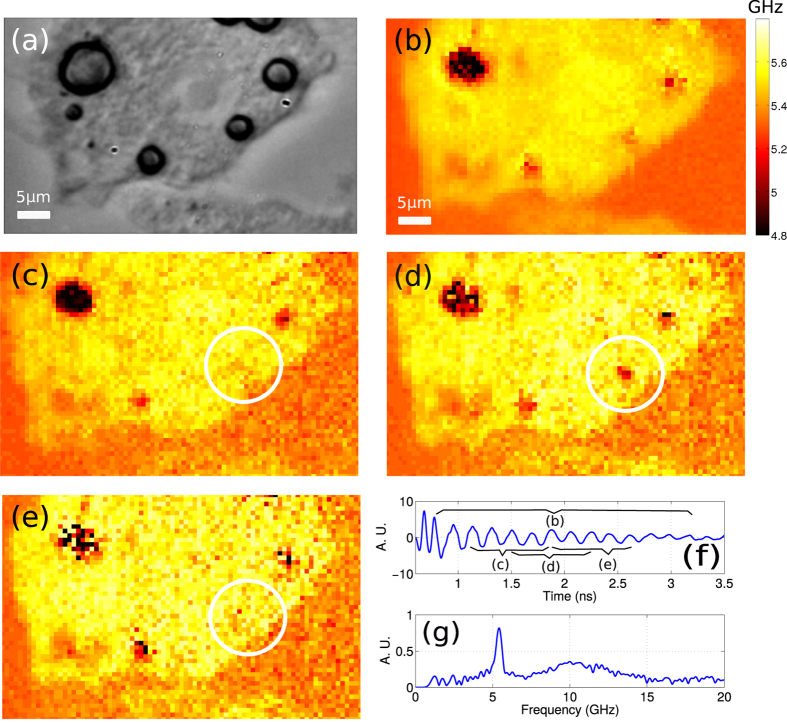
Imaging of an adipose cell using ~5 GHz phonons. (**a**) Optical picture taken with a conventional brightfield microscope showing an adipose cell. Fat droplets are clearly visible. (**b**) The Brillouin frequency map of the area shown in (**a**). This map was obtained using the complete temporal extent of the detected signals. (**c**–**e**) Subsections of the measured volume. The central position of the windows in the z direction are 0.6,1 and 1.4 *μ*m to figures (**c**–**e)** respectively. The fat droplet marked with a circle appears and disappears within the measured volume. (**f**) Typical time trace observed from the cell cytoplasm shown as a star in (**a**). (**g**) Fourier transform of the time trace presented in (**f**) showing the Brillouin frequency peak.

Cells can be expected to show mechanical contrast between stiff, aligned structures such as the nucleus or sarcomeres compared to the cytoplasm. In the case of the imaging modality presented here, the sound is detected with Brillouin scattering where stiff structures reveal themselves with a higher sound velocity (and therefore higher *f*_*B*_) than soft structures. Figure 5 shows an example of our approach applied to a fixed adipose cell cultured on our transducer with a glass substrate. The acoustic wavelength in this case is approximately 280 nm if a refractive index of 1.36 is assumed^38^. A brightfield image of the scanned area is shown in Fig. 5(a). The Brillouin frequency map shown in Fig. 5(b) was obtained by calculating the Fourier transform over the complete temporal extent of the acquired signal. The fat droplets show distinct contrast compared to the rest of the cell. This contrast is expected as fat has different mechanical properties from the rest of the cell. By assuming a refractive index it is possible to convert the temporal axis to a spatial axis (z). By doing so and sectioning spatially the measurement of the Brillouin frequency with *N*_*λ*_ = 4, three critically sampled z sections with resolution of 560 nm were obtained. Such sections are shown in Fig. 5(c–e). From there it is clear to see that the fat droplet marked with a black circle appears in the second section and disappears in the third. This shows that the object is ~560 nm in height and its position is near the centre of the second section (~1 *μm*) as there is little influence from it on the other sections.

### Live cell imaging

Imaging living cells using laser-generated phonons requires the specimen to survive exposure to the light and heat of the system and the imaging time must be as short as possible to prevent motion artifact and capture dynamic processes. The damage threshold of cells caused by near-infrared (NIR) laser pulses were reported to be safe for power densities of 1.7 × 10^14^ W/m^2^ scanning a sub-micron spot for over 35 minutes^39^. In our measurements the power density is approximately 8.5 × 10^12^ W/m^2^ scanning a one micron spot for ~38 minutes which is comparable to what has been reported as safe. It is well known that UV light is particularly harmful to cells, however, damage thresholds for single cells using short 390 nm pulses have not been reported in the literature and are anecdotally low and to be avoided if at all possible. Using our schema the sample receives a typical reduction of probe (5x) and pump (15x) beam intensity for the same input power compared to previously reported phonon detection methods^22^,^32^. While beam exposures are thus minimised by the transducer and measurement geometry, thermal exposure remains a problem when using glass as substrate due to the low thermal conductivity of the substrate. Numerical models (see supplementary information) show the steady state temperature rises at the cell/substrate interface to be around 25 °C above room temperature (20 °C) when a glass substrate is used. This is due to absorption of the laser pulse train and poor thermal conduction. Such temperatures can be fatal to cells, particularly if heat accumulates over the large numbers of measurements needed to build images using phonons. Sapphire, which has ~30 times the thermal conductivity of glass, was used as a substrate with living cells to minimise interfacial temperature rises to ~7 °C above room temperature. Managing the thermal load during imaging restricts temperature rise to the physiological tolerable range, allowing acquisition of thousands of measurements without compromising cell viability.

**Figure 6:**
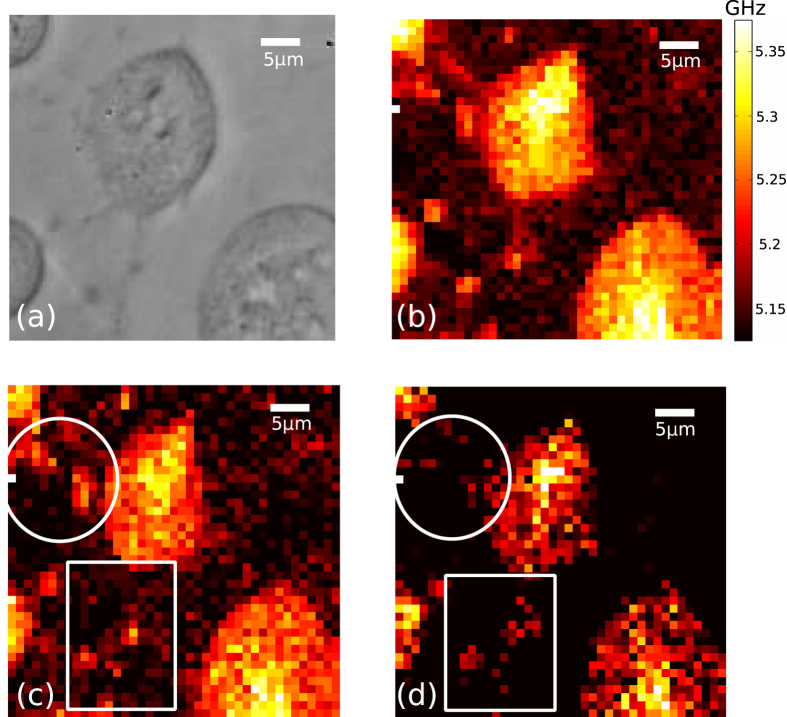
Imaging of live 3T3 cells with phonons. (**a**) Optical image of the scanned area. (**b**) Brillouin shift measured from (**a**) sampling every 1 *μ*m, taking ~1.5 s per point and a total of 38 minutes total acquisition time. (**c**) Section obtained at d = 1 *μ*m. (**d**) Section obtained at d = ~1.8 *μ*m. As sound propagates, thin filopodia (marked by white circle, square) are resolved axially as the section moves deeper into the cell.

Figure 6 shows an example of live cell imaging (3T3 mouse fibroblasts) using a sapphire substrate and 280 nm wavelength phonons. There is good visual agreement between the optical (Fig. 6(a)) and ultrasonic (Fig. 6(b)) images. The average power applied to the transducer was 0.4 and 1 mW for pump and probe beams, of which the cell receives ~0.04 and 0.3 mW respectively. Our methods thus irradiates the sample with far less optical power than required in other phononic (~7.5 mW pump and 2.2 mW probe^32^) and Brillouin microscopy (2–5 mW^17^,^18^) approaches. To confirm compatibility with living cells, the state of the cells was dynamically assessed through the presence of propidium iodide (PI) in the bathing medium. PI fluoresces strongly in the red when bound to DNA, from which it is excluded by the cell membrane in living cells. Continued exclusion of PI throughout the experiments showed that cells remained alive throughout and after the imaging process had finished. Adding detergent to the solution post-experiment killed the cells and confirmed the presence and function of PI (see supplementary information). This demonstrates that the thermal load is a dominant damaging factor and that the characteristics of our approach have provided adequate protection against thermal and photon damage.

Sectioning of living cells was also possible. By selecting *N*_*λ*_ = 6 for 840 nm resolution, contrast is obtained along the z axis (see Fig. 6(c,d)). Filopodial features are shown to be thinner than the axial section (Fig. 6 circle, square), thus morphology and extent are seen to change as the section is shifted through the cell.

**Figure 7:**
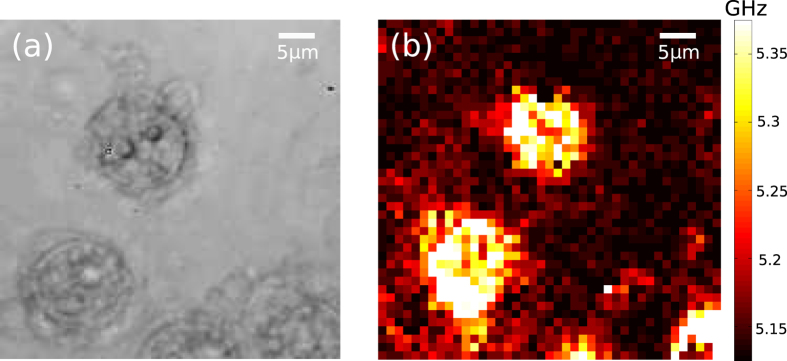
Imaging of delipidated cells using phonons. (**a**) Brightfield image of 3T3 fibroblast cells. (**b**) Map of the Brillouin shift obtained from the cell presented in (**a**). The appearance of the cells is considerable different than the living cells shown in Fig. 6, their range of acoustic frequencies is also significantly larger.

Previous reports of cell imaging using phonons have historically been performed in treated cells (fixed and/or dehydrated). The use of detergent to kill the cells post-experiment offered further opportunity to assess the mechanical contrast of cells whose membranes are delipidated by the detergent. Figure 7 shows cells that were deliberately killed using detergent which changed their appearance not only optically but mechanically as well (see Fig. 6 for comparison). Higher frequencies are observed in the dead cells compared to the living. These higher frequencies arise possibly because when the cell collapses due to the dissolution of its lipid membrane, all the stiff structural material is packed closely together.

## Discussion

This paper has introduced label free high resolution imaging of living cells using phonons. This technique has the ability to axially resolve structures based on mechanical contrast intrinsic to the sample with better than optical resolution. By implementing an optimised transducer design to protect the sample from thermal and photon damage, we further show the method to be compatible with live cell imaging. This technique offers a label-free alternative for live-cell imaging and mechanical characterisation.

The contrast in the images depend on the optical and mechanical properties of the sample. For the types of cells presented in this paper the change in the measured Brillouin frequency within a cell is predominantly related to changes in the acoustic velocity as the required change in refractive index to cause the observed frequency shift would be many times greater than that observed previously^40^. Combining the present technique with an optical method for locally measuring the refractive index would allow the velocity to be recovered directly, this is possible as the all optical nature of the instrument allows combination with other imaging modalities.

It is interesting to note that the contrast seen in living cell images is lower than for fixed cells^24^, There are a number of potential reasons for this observation. Firstly, the fixing process of cross linking internal structures with polymers could make the fixed cells appear stiffer and hence faster then their living counter parts. Secondly, it is possible that some finer structures in living cells are moving (such as filopodia), however as single point is acquired in 1–2 seconds it is unlikely that this would have a big impact on the measured contrast. It would however have an impact on the spatial correlation of fine features within the final image as the time taken between adjacent points can be significant and it is possible that the movement of these features could introduce artifacts. A significant increase in imaging speed could be achieved by recording signals in parallel, this has been achieved for non-ASOPS pump probe measurements^41^, a modified scheme could be applied here. With faster image acquisition rates images of dynamic cells or processes could be obtained.

In principle the highest axial resolution using the phononic imaging technique presented here is *λ*_*probe*_/2*n*, which is ~280 nm in cells at *λ*_*probe*_ = 780 nm. This is significantly smaller than the optical sectioning capabilities of the optical system used to take the measurements, (which with an NA of 0.42 has a depth of focus of approximately 8 microns) and is higher than that achievable with a typical oil immersion confocal system. In practice the axial resolution is currently limited by noise to 560 nm and 840 nm for the fixed and living cells respectively.

In contrast to most other sectioning techniques time resolving the acoustic signal allows the sectioning information to be acquired in one go, there is no need to re-scan the sample for each section required. The sections are recovered in post processing and the cell therefore only receives one dose of light to obtain all the information in the 3D volume. This gives flexibility in the processing as a high SNR image of the average velocity can be recovered if the entire signal is used or separate sections can be recovered to show more detail. The time to acquire the signal is the same regardless, which is ~1–2 seconds per point, which typically yields 5–10 voxels per point if the data is sectioned.

The lateral resolution is currently limited by the size of the optical spots used to generate and detect the ultrasound. It is possible to make the size of the acoustic spots smaller than the optical spots in a number of ways, for example, by engineering the spatial properties of the thin film substrate or by using nanoparticles to generate sound waves with sub optical resolution in all directions, but this requires further research. There is much scope to take advantage of the broad bandwidth available from laser-generated phonons (>100 GHz, with *λ* *~* 15 nm) in the search for a very high resolution imaging tools.

## Methods

### Experimental set-up

The asynchronous optical sampling (ASOPS^27^) pump probe system (see Fig. 2) controls two 150 femtosecond pulsed lasers with repetition rates of ~100 MHz and allows the delay between the lasers to be set and swept electronically without the need for a mechanical delay line^21^,^22^. In our system, the delay repetition rate between the probe and the pump is 10 kHz which means that a measurement (of the complete 10 ns delay sweep) is taken every 100 *μ*s.

As seen in Fig. 2, both beams (*λ*_*probe*_ = 780 *nm, λ*_*pump*_ = 390 *nm*) are combined and focused together through a 50x (NA = 0.55) long working distance objective. Adjustable mirrors allows coaligning of the pump spot to the probe spot. The probe beam is detected after being collected by an objective lens (20x, NA = 0.42) and focused to a photodiode. The sample is scanned by moving electromechanical stages with a minimum step motion of 100 nm. Typically 10000 averages are taken per point which takes ~2 s to acquire limited by data acquisition element. The system uses typical average powers of 1 mW (0.3 mW at cell) in the probe and 0.5 mW (0.05 mW at cell) in the pump corresponding to pulse energies of 10 pJ and 5 pJ and peak powers of ~60 W and ~30 W respectively.

Optical imaging is performed with two cameras: one CCD is used for brightfield imaging (using the 50x objective lens) and the other (emCCD) for fluorescence (using the 20x objective lens). A spectrally-distinct LED source (530+/−25 nm) provides brightfield illumination of the sample as well as excitation for red-emitting fluorescence dyes. Epifluorescence was detected via a TRITC cube (Ex: 535/15, DCX: 565, Em: 615/35 nm).

### Cell methods

3T3 fibroblast cells were cultured on the EtOH-sterilised transducers for 24 hrs in standard culture medium. Living cells were kept in Hanks balanced salt solution (HBSS) buffered with 4-(2-hydroxyethyl)-1-piperazineethanesulfonic acid (HEPES, 25 mM) to maintain physiological pH (7.4) under ambient conditions during the experiments. Media was pumped at 0.05 *μ*Ls^−1^ using a syringe pump into a chamber (chamilde CF-T25 produced by Live Cell Instruments, Seoul) comprising a transducer substrate and a closing coverslip. Constant flow of media allowed specimens to remain alive for several hours at room temperature (21 °C).

A fluorescence assay was used to confirm cell viability (Fig. 6). Propidium iodide (PI, abs: 510–560 nm, em: 600–650 nm) binds to DNA in the nuclei of dead cells, and is non-fluorescent when excluded from entering living cells by the intact cell membrane. PI was added as a 1 mM EtOH stock to a concentration of 1 *μ*M in bathing medium. A fluorescence picture of the targeted region of cells was obtained before starting the ultrasonic imaging and another following the ultrasound imaging experiment (see supplementary Fig. 1). Finally, all cells were deliberately killed using a detergent (Triton X-100 in phosphate buffered saline (PBS) solution, Sigma-Aldrich). Cells which survived the ultrasound imaging experiments continued to exclude PI from their nuclei throughout and following the experiment, confirming that the cells remained viable (see supplementary information).

### Signal processing

Raw signals are composed of several parts: coincidence peak, thermal response and signals of interest - which were extracted by established signal processing^24^,^33^. A fast Fourier transform (FFT) is then performed on the resultant trace to measure the Brillouin frequency. Signal to noise ratio (SNR) was evaluated in the frequency domain by measuring the peak amplitude of the acoustic signal with the standard deviation of the noise background in the absence of signal (pump off) and in the same band.

Because the acoustic waves are propagating axially through the sample, different moments in time represent different locations in space. Axial sectioning information was therefore obtained by resolving the Brillouin frequency in time. This was possible using the short time Fourier transform method (STFT) where a small time window (length: two acoustic cycles or more) is shifted in time. In this way, it is possible to section the optical volume. The resolution of this process was estimated by modelling and experimental measurements of an edge response revealing the axial resolution is approximately half of one measuring window when the window lasts for two cycles or more.

## Additional Information

**How to cite this article**: Pérez-Cota, F. *et al*. High resolution 3D imaging of living cells with sub-optical wavelength phonons. *Sci. Rep.* **6**, 39326; doi: 10.1038/srep39326 (2016).

**Publisher's note:** Springer Nature remains neutral with regard to jurisdictional claims in published maps and institutional affiliations.

## Supplementary Material

Supplementary Information
