# Supplementary material for: High resolution 3D imaging of living cells with sub-optical wavelength phonons
Source: Sci Rep. 2016 Dec 20;6:39326. doi: 10.1038/srep39326 (PMC5171858; doi:10.1038/srep39326)
Supplement: Supplementary Information [file srep39326-s1.pdf]

Supplementary information for "High resolution 3D imaging of living cells with sub-optical wavelength phonons"

Fernando Pérez-Cota,* Richard J. Smith, Emilia Moradi, Leonel Marques, Kevin F. Webb and Matt Clark

Optics and photonics group, University of Nottingham,

University Park, Nottingham, UK

*ezzfp@nottingham.ac.uk

November 16, 2016

Live/dead fluorescence assay

Propidium iodide (PI) is a red-emitting fluorophore which becomes fluorescent when bonds to DNA inside cells. It is membrane impermeable, and therefore excluded from viable cells. In the presence of PI, cell death results in the transmembrane entry of PI, which binds to the DNA and causes fluorescence within the nucleus - thus confirming death of the cell. Supplementary Fig. 1 shows the result of the assay performed for the cell presented in Fig. 3 as described in the methods. Supplementary Figs. 1b and 1c show fluorescence before and after the experiment respectively where no changes in nucleus fluorescence are observed. After the experiment has finished, the cells are exposed to detergent to kill them (Triton X-100, 2%). Supplementary Fig.1d shows that the cells are labelled with PI, and therefore killed, only after detergent was applied- thus demonstrating that the cell remained alive before, during and after the experiment using high-frequency ultrasound.

Thermal exposure

Using a simple finite element (FE) thermal-only model that consisted of a geometry, based on cylindrical coordinates, of substrate-transducer and aqueous media (see Fig. 2) it was possible to estimate the temperature rise in the cell/transducer interface for a single pulse (transient) and for a given average power (steady state and transient) over time. To mimic the composition of a biological cell, the thermal properties of water were used. The parameters for all materials are displayed in Table 1. The model was then used to solve the heat equation:

$$\rho C_p \frac{\delta T}{\delta t} - \nabla(k \nabla T) = Q \quad (1)$$

where ρ is the density of the material, C_p the heat capacity, k the thermal conductivity, T the temperature, t the time, ∇ the Laplace operator and Q the heat flux. The initial conditions

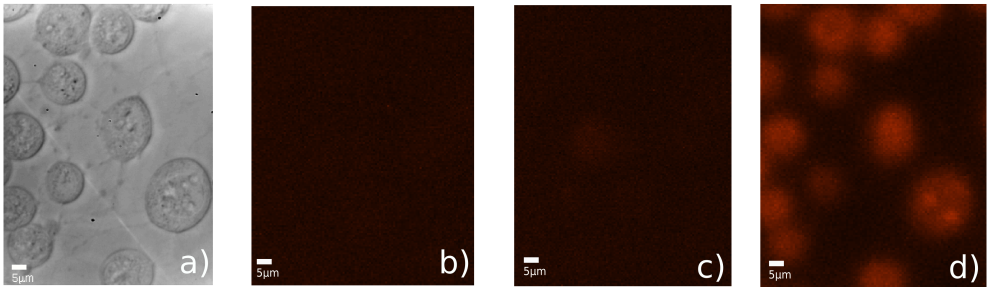

Supplementary figure 1: Live/dead assay based on the entry of propidium iodide into dead cells. a) Brightfield image of 3T3 fibroblast cells, showing cells imaged using our approach. b) Fluorescence image before the experiment. b) Fluorescence of same area after the ultrasonic experiment was finished - no fluorescence is observed indicating cell survival throughout. d) Positive control fluorescence image of the same area after the cells were exposed to detergent showing strong fluorescence of the cell nuclei following treatment with Triton X-100.

Material	Thermal conductivity$Wm^{-1}K^{-1}$	Heat capacity $JKg^{-1}K^{-1}$	Density Kgm^{-3}
Silica Glass	1.38	703	2203
Sapphire	35	730	3965
Gold	317	129	19300
ITO	10.2	700	7140
Water	0.6	4180	998.3

Table 1: Thermal properties of materials used in FE models.

for the substrate and the water are Q_0=0, but for the transducer layers for average power (transient and steady state):

$$Q_0 = \frac{P_{Avg}}{\pi W_0^2 \delta_p} exp(-z^2/W_0^2) \tag{2}$$

and for a single pulse (transient only):

$$Q_0 = \frac{P_{peak}}{\pi W_0^2 \delta_p} exp(-z^2/W_0^2) \frac{exp(-t^2/t_0^2)}{t_0} t \tag{3}$$

where P_{avg} and P_{peak} are the average and peak power absorbed by each layer respectively, W_0 the focal spot radius, t_0 the pulse width and δ_p the skin depth for all layers which in this case corresponds to the thickness of each layer given that all the layers are thinner than their corresponding skin depth. The first exponential on both equations 2 and 3 represents the change in intensity as given by the laser spot, while the second exponential in Eq. 3 represents the change in intensity over time given by a single pulse. The boundary conditions were set as shown in Fig. S2 with axial symmetry for the inner boundaries and a set temperature (T=20°C) for the surrounding ones.

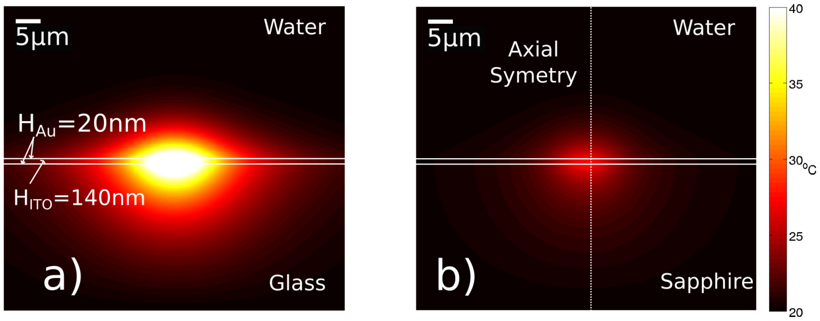

Supplementary figure 2: Steady state temperature distribution due to pump absorption. (a) Temperature distribution for a 1mW of average pump power using glass substrate. (b) Same using a sapphire substrate. A reduction of 20^oC at the cell/substrate interface is achieved by using a sapphire instead of a glass substrate. Geometry used for the FE model (gold layers shown as white lines) and boundary conditions are shown.

The steady-state analysis for average power shown in supplementary Fig. 2 shows that for the same average input power (1mW, which is partially absorbed by the layers), there is a reduction of the temperature at the water/transducer interface of approximately 20°C when changing the substrate material from glass to sapphire. At room temperature, the temperature deviation observed using sapphire is thus safe for biological specimens (T_{max} ∼27°C) unlike the case when a glass substrate is used (T_{max} ∼47°C).

The thermal dynamics at the surface of substrates irradiated by our standard imaging conditions is shown in supplementary Fig. 3. In the single-pulse case (Fig. 3a), is possible to see that the temperature rise at the transducer/cell interface(solid blue line) is smaller than the one for the transducer/substrate interface (solid red line). However, as time goes on, the temperature of both surfaces becomes the same which implies that thermally having the cell away from the main heat source (first gold layer) does not reduce heat exposure significantly. For the case of continuous pulses (at 0.4mW pump average power), (Fig. 3b), the system reaches thermal equilibrium after approximately 5μs. This time is short compared with the acquisition of a single trace (100μs) which means it is possible to assume that the temperature distribution obtained from the steady state calculations is correct for every measurement.

Fundamentals of Brillouin scattering

From a classical point of view, a propagating compressional wave generates a change of refractive index where the compression takes place due to the opto-elastic effect. The wave then generates a refractive index grating with a period that equals the acoustic wavelength. An incident optical beam will interfere constructively producing a strong reflection of a particular wavelength. The wavelength that satisfies the Bragg condition is given by:

$$\lambda_{Bragg} = 2n\Lambda cos\theta \tag{4}$$

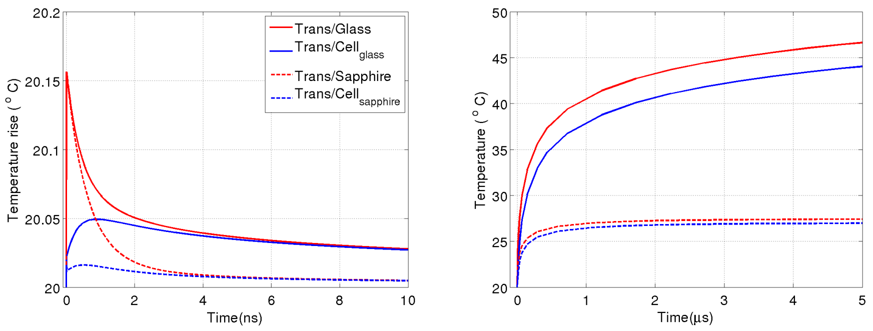

(a) (b)

Supplementary figure 3: Temperature rise due to pump absorption. Red lines represent the transducer/substrate interface and Blue lines the transducer/cell. Solid lines represent glass substrate while dotted represent sapphire. In both cases there is a reduction of the instantaneous temperature rise on the cell/transducer interface compared with the transducer/substrate one. By the end of the measuring window (10ns), the temperature on both interfaces is the same. Sapphire however shows greater heat dissipation which may benefit reduction of living cells. a) Temperature distribution for a single pulse (fast time scale). b) Temperature rise for a continuous train of pulses (slow time scale).

where n is the refractive index of the medium, Λ the period of the grating and θ the optical incident angle. Since the period can be written in terms of a the speed of sound ν and the acoustic frequency f as $\Lambda = \nu/f$, the Bragg condition can be rewritten as:

$$\lambda_{Bragg} = 2n\frac{\nu}{f}cos\theta \tag{5}$$

Then the acoustic frequency that will scatter a particular wavelength λ is known as the Brillouin frequency f_B which at normal incidence is given by:

$$f_B = \frac{2n\nu}{\lambda} \tag{6}$$

The acoustic wavelength of the probed acoustic wave (λ_a) is then given by:

$$\lambda_a = \frac{\nu}{f_B} \tag{7}$$

so then:

$$\lambda_a = \frac{\nu}{\frac{2n\nu}{\lambda_{probe}}} = \frac{\lambda_{probe}}{2n} \tag{8}$$

The probed acoustic wavelength (and thus resolution) then depends only on optical parameters because the scattered light is the one that fulfils the Bragg condition. If only the sound velocity changes, then a different acoustic frequency is probed by the same optical wavelength.

In a time-resolved measurement of Brillouin scattering, the sound propagates away from the generation point along the z-axis. As it does so, it scatters light from different spatial positions in the material. If the refractive index is known, then it can be possible to calculate the speed of sound as:

$$\nu = \frac{\lambda_{probe} f_B}{2n} \tag{9}$$

allowing to convert temporal axis in spatial axis as:

$$z = t\nu \tag{10}$$

which allows to resolve the speed of sound of along the z axis with the acoustic wavelength rather than the optical. There are a number of methods to resolve in time frequency of a given signal. The method we have chosen is short time Fourier transform (STFT). This processing method performs Fourier transforms with short time windows instead of whole traces. This allows the detection of different frequencies as the widow advances in time.

Modelling

Thermo-elastic generation of phonons can be modelled by the theory described by Matsuda et al**?**. This model was implemented to produce simulated signals that allowed to analyse the resolution of the presented method. In it, the thermal stress induced by the absorption of an optical pulse (pump) and its propagation through a sample with multiple layers is evaluated. Such stress can interact with a second light beam (probe) in a number of ways. For instance, surface displacement can be calculated directly form stress. This is appropriate for sound detection methods that are sensible to surface displacement (see Fig. 4).

For our particular application, evaluation of the refractive index modulation induced by stress is adequate to model Brillouin oscillations if the medium is transparent. Temperature also modulates refractive index however this is not included in our model. Figure 4 shows a simplified flow diagram of how the model works. The red lines indicate the path followed to obtain our modelled signals.

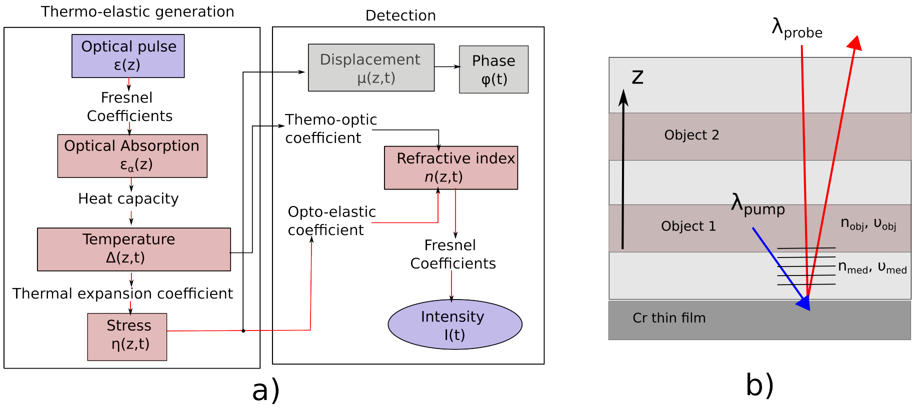

(a)

Supplementary figure 4: Flow diagram of phonon generation/detection model. The diagram shows simplified steps for the calculation of stress induced by an optical pulse and its influence on a probing beam.
